# Simultaneous Evaluation of Bone Cut and Implant Placement Accuracy in Robotic-Assisted Total Knee Arthroplasty

**DOI:** 10.3390/jcm13051293

**Published:** 2024-02-25

**Authors:** Killian Cosendey, Julien Stanovici, Hugues Cadas, Patrick Omoumi, Brigitte M. Jolles, Julien Favre

**Affiliations:** 1 Department of Musculoskeletal Medicine, Lausanne University Hospital and University of Lausanne (CHUV-UNIL), CH-1011 Lausanne, Switzerland; killian.cosendey@chuv.ch (K.C.); brigitte.jolles-haeberli@chuv.ch (B.M.J.); 2 Morphology and Anatomy Faculty Unit, University of Lausanne (UNIL), CH-1005 Lausanne, Switzerland; hugues.cadas@unil.ch; 3 Department of Diagnostic and Interventional Radiology, Lausanne University Hospital and University of Lausanne (CHUV-UNIL), CH-1011 Lausanne, Switzerland; patrick.omoumi@chuv.ch; 4 Institute of Electrical and Micro Engineering, Ecole Polytechnique Fédérale de Lausanne (EPFL), CH-1015 Lausanne, Switzerland; 5 The Sense Innovation and Research Center, CH-1007 Lausanne, Switzerland

**Keywords:** total knee arthroplasty, robotic-assisted surgery, accuracy, bone cuts and implant placements, position and orientation errors

## Abstract

**Background:** This study aimed to evaluate the accuracy of bone cuts and implant placements, simultaneously, for total knee arthroplasty (TKA) performed using a system with an active robotic arm. **Methods:** Two experienced orthopaedic surgeons performed TKA on ten cadaveric legs. Computed tomography scans were performed to compare the bone cuts and implant placements with the preoperative planning. The differences between the planned and actual bone cuts and implant placements were assessed using positional and angular errors in the three anatomical planes. Additionally, the cut–implant deviations were calculated. Statistical analysis was performed to detect systematic errors in the bone cuts and implant placements and to quantify the correlations between these errors. **Results:** The root-mean-square (RMS) errors of the bone cuts (with respect to the planning) were between 0.7–1.5 mm and 0.6–1.7°. The RMS implant placement errors (with respect to the planning) varied between 0.6–1.6 mm and 0.4–1.5°, except for the femur and tibia in the sagittal plane (2.9°). Systematic errors in the bone cuts and implant placements were observed, respectively, in three and two degrees of freedom. For cut–implant deviations, the RMS values ranged between 0.3–2.0 mm and 0.6–1.9°. The bone cut and implant placement errors were significantly correlated in eight degrees-of-freedom (ρ ≥ 0.67, *p* < 0.05). **Conclusions:** With most of the errors below 2 mm or 2°, this study supported the value of active robotic TKA in achieving accurate bone cuts and implant placements. The findings also highlighted the need for both accurate bone cuts and proper implantation technique to achieve accurate implant placements.

## 1. Introduction

Robotic assistance is very promising in total knee arthroplasty (TKA), specifically to achieve accurate bone cuts and thus increase the accuracy of implant placements. This is particularly important because a more accurate placement of implants has been suggested to improve patient-reported outcomes [1,2,3]. Such benefits, in comparison to conventional surgery, have already been reported with some robotic-assisted systems, notably improvements in implant placement accuracy, increases in patient satisfaction, and/or reductions in complications [3,4]. Nevertheless, not all robotic systems are necessarily equally effective. Therefore, there is a need to assess the systems and their new releases continuously to ensure that they are used properly and possibly also to highlight areas of improvement.

Over the years, diverse approaches to TKA robotic assistance have been proposed, some moving surgical tools and others monitoring the movement of the surgical tools moved by the surgeons. Differences also exist in the means of cutting the bone, with some solutions using a saw and others using a cutter. One interesting option is the TSOLUTION ONE^®^ Total Knee Application (“TSOLUTION ONE” in this article, THINK Surgical Inc., Fremont, CA, USA). It includes a surgery device with a robotic arm controlling a cutter (named TCAT^®^) and a surgical planning workstation (named TPLAN^®^). It differentiates itself from the other options currently on the market by the fact that it is the only system that actively cuts the bones. So far, this system has been reported to cut the bones with root-mean-square errors below 2 mm and 1° in six degrees of freedom for both the femur and the tibia [5]. The root-mean-square errors in implant placements within 1.5 mm and 1.5° have also been reported for eight degrees of freedom, including the femoral anterior–posterior position and internal–external rotation angle, as well as the femoral and tibial proximal–distal position, flexion–extension and varus–valgus angles [6]. While these previous studies provide important insights, they differ on several aspects, which prevents the combination of their results, for example, to assess the deviations between the bone cuts and the implant placements. Indeed, in addition to differences in implant types and error calculation methods, one study analysed implant placements in human knees [6], whereas another assessed bone cuts using sawbone knees [5]. Consequently, additional studies simultaneously assessing the accuracy of bone cuts and the accuracy of implant placements are needed for the TSOLUTION ONE system. Evaluating the deviations between bone cuts and implant placements is particularly motivated by previous studies on manual TKA, showing that implants are not always placed perfectly in contact with the cut surfaces of bones [7,8,9] and by the fact that the deviations could be different with bones cut by a robotic arm rather than manually. 

The purpose of this study was to assess the accuracy of bone cuts and implant placements simultaneously for TKA performed using the TSOLUTION ONE system. The study also aimed to evaluate the cut–implant deviations.

## 2. Materials and Methods

Following approval from the local ethics committee, TKA was performed on 10 formalin-fixed, anonymized cadaveric legs using the TSOLUTION ONE system (version 300). The sample size was determined based on previous comparable studies and ethical considerations [10,11,12]. According to local regulations regarding research on deceased persons, no demographic data were available for the samples. The procedures were performed by two senior orthopaedic surgeons from our university hospital, with more than 5 years of independent TKA practice. The surgeons, who were previously trained on the system using sawbones and cadaveric knees, conducted, respectively, four and six cases, following the manufacturer’s recommendations for regular TKA on patients. To increase reliability, all data acquisition and processing were conducted by a single operator [13]. The 6-step protocol used for each study knee is described below.

Step 1: five fiducial markers (titanium beads of 0.8 mm diameter) were embedded in the femur and tibia to allow them to register the original bones (before cutting) with the cut bones in step 4.

Step 2: Preoperative CT images of the cadaveric leg were acquired with a Discovery CT750 HD machine (GE Healthcare, Chicago, IL, USA) parametrised as follows: field of view of 250 × 250 mm, matrix size of 512 × 512 pixels, tube voltage of 120 kVp and tube current of 200 mAs. Two different slice thicknesses were used: high resolution of 0.312 mm and low resolution of 0.625 mm. After uploading the low-resolution CT images in the surgical planning software, the three-dimensional (3D) surface models of the bones were reconstructed and used to plan the TKA. For this study, all procedures were carried out with the “Unity Posterior Stabilized Femoral” implant and the “Unity Tibial” implant (Corin, Cirencester, UK). Once the planning was completed by the surgeon, it was transferred to the robotic device.

**Figure 1:**
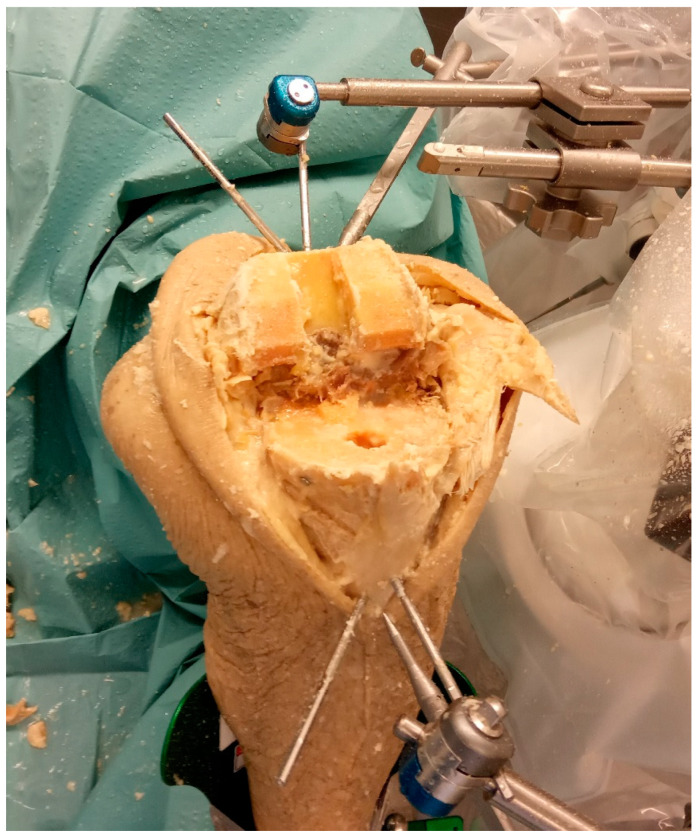
Example of a study knee after bone cutting.

Step 3: The preparation and calibration of the robot followed the standard procedure of any TKA intervention on patients. Next, the surgeon exposed the cadaveric knee following a medial parapatellar approach. Following the instructions on the robot’s screen, the surgeon then recorded the position of registration points on the surface of the bones using a mechanical digitizer. After that, the robot registered the 3D surface model of the bones on the actual bones and cut the femoral and tibial bones according to the planning (Figure 1). The bones were cut autonomously by the robot, under the surgeon’s supervision. Once the bone cuts were completed, nylon implants were impacted and cemented by the surgeon who then sutured the leg following the usual procedure. Nylon implants were used to avoid the artefacts in the CT images induced by the metal of regular implants particularly affecting the bone (cut) close to the implants [14,15].

Step 4: Postoperative CT images of the cadaveric leg were acquired using the same high-resolution parameters as for the preoperative CT scan described in step 2. Based on these images, the 3D surface models of the cut bones and of the implants were reconstructed using in-house software. Then, the cut bone models and the implant models were imported in the preoperative CT frame and registered on the preoperative data (original bone models and planning). Registrations were carried out separately for the tibia and the femur by locating the centre of the fiducial markers and calculating the mathematical transformation, mapping the markers of the cut bones to the markers of the original bones [16].

Step 5: To quantify the accuracy, cut and implant frames using the same definition as the planning frames were embedded in the 3D surface models of the cut bones and in the 3D surface models of the implants. Then, the differences between the planning frames and the cut frames as well as between the planning frames and the implant frames were calculated as three positional and three angular errors. The errors were expressed in the anatomical frames proposed by Victor et al. [17]. Additionally, the cut–implant deviations, defined as the difference between the implant placement errors and the bone cut errors, were calculated independently for each degree of freedom. By convention, positive femoral errors indicate a cut or an implant too lateral, proximal, anterior, varus, internally rotated and extended compared to the planning or compared to the cut. Tibial errors were defined similarly to the femoral errors, except in the coronal and sagittal planes, where positives errors indicate a cut or an implant too flexed and valgus compared to the planning or compared to the cut. Since the internal–external rotation of the tibia implant was not constrained by the bone cuts, no error was calculated for this degree of freedom.

To assess the reliability of the error measurement method, two randomly selected knees were CT-scanned and processed five times each by the single operator in this study. This procedure indicated root-mean-square (RMS) differences among repeats under 0.2 mm and 0.3° for all positional and angular errors. 

### Statistical Analysis

Bone cut errors, implant placement errors and cut–implant deviations were reported through their median, interquartile range (IQR) and root-mean-square (RMS). In adequacy with a study population limited to 10 knees, data were tested using non-parametric statistics. Specifically, systematic errors (i.e., biases) were detected using Kruskal–Wallis tests, followed by post hoc Wilcoxon signed-rank tests with Bonferroni corrections. Spearman’s rho correlations were calculated between bone cut and implant placement errors to estimate the influence of the bone cut errors on the implant placement errors. The significance level was set a priori to 5%. In addition, the number of knees presenting outlier errors or deviations (defined as errors or deviations exceeding ± 3 mm or ± 3°) was recorded [18]. Data processing and statistical analysis were performed with Matlab R2019b (Mathworks, Natick, MA, USA). 

## 3. Results

**Figure 2:**
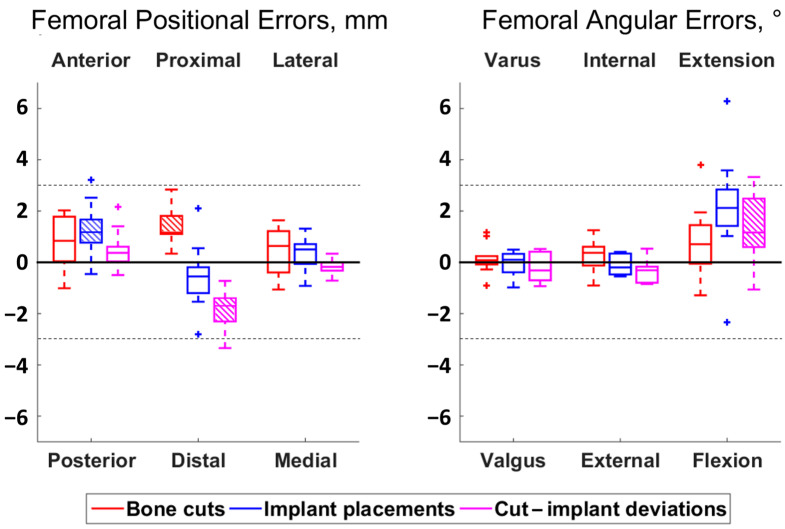
Boxplot of the positional (**left**) and angular (**right**) errors and deviations for the femur. Each boxplot displays the interquartile range (box), median value (line), and outliers (crosses). The directions above and below the plots indicate where the actual cuts or implant placements were compared to the planning or compared to the cuts. Hatched boxes indicate errors or deviations statistically significantly different from zero (i.e., with a bias) (adjusted *p* < 0.05). Dashed lines are included at ±3 mm and ±3° to delineate the outlier thresholds.

**Figure 3:**
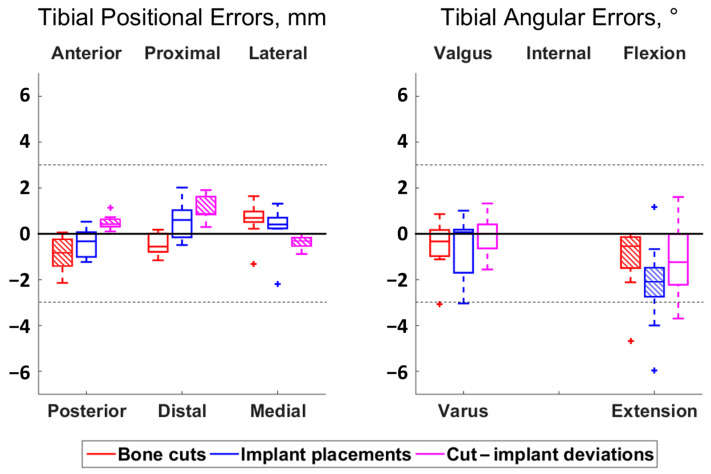
Boxplot of the positional (**left**) and angular (**right**) errors and deviations for the tibia. Each boxplot displays the interquartile range (box), median value (line), and outliers (crosses). The directions above and below the plots indicate where the actual cuts or implant placements were compared to the planning or compared to the cuts. Hatched boxes indicate errors or deviations statistically significantly different from zero (i.e., with a bias) (adjusted *p* < 0.05). Dashed lines are included at ±3 mm and ±3° to delineate the outlier thresholds.

Errors and deviations are reported using boxplots in Figure 2 and Figure 3, whereas numbers are provided in Appendix A (Table A1 and Table A2).

Regarding the bone cut errors, the RMS values ranged between 0.7–1.5 mm and 0.6–1.7°. Three cases of bias were observed: in the femoral proximal–distal position (*p* = 0.01), with cuts too proximal compared to the planning (median error of 1.2 mm), and in the tibial antero–posterior position (*p* = 0.01) and flexion–extension angle (*p* = 0.01), with cuts too posterior and extended compared to the planning (median errors of 0.8 mm and 0.5°, respectively). In total, 3 of the 110 individual measurements exceeded the ±3 mm or 3° thresholds and were considered outliers.

The RMS errors of the implant placements varied between 0.6–1.6 mm and 0.4–1.5°, except in the femoral and tibial flexion–extension angles, where the errors had RMS values of 2.9°. Two cases of bias were observed: in the femoral anterior–posterior position (*p* = 0.02), with implants too anterior compared to the planning (median errors of 1.2 mm), and in the tibial flexion–extension angle (*p* = 0.02), with implants too extended compared to the planning (median errors of 2.1°). A total of 6 outlier errors were observed, from the 110 individual measurements.

**Figure 4:**
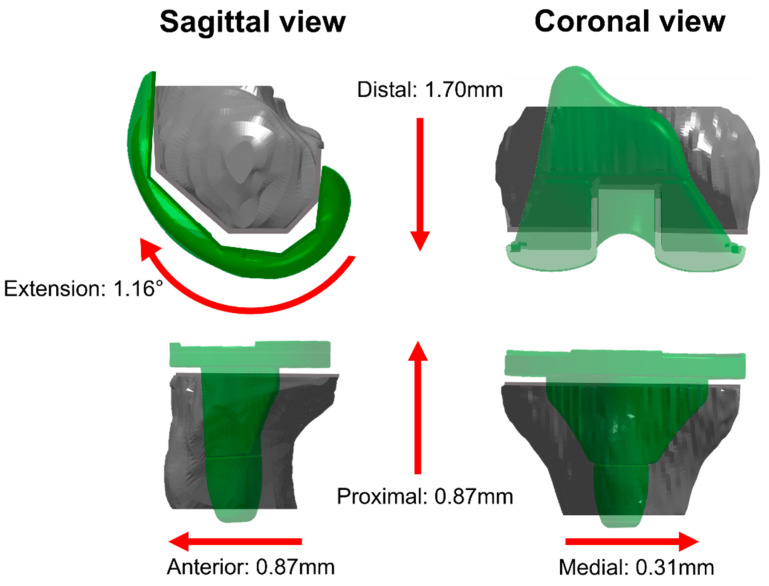
Illustration of the biases in cut–implant deviations for a right knee. The red arrows represent the systematic deviations (median values) observed on the 10 study knees.

**Figure 5:**
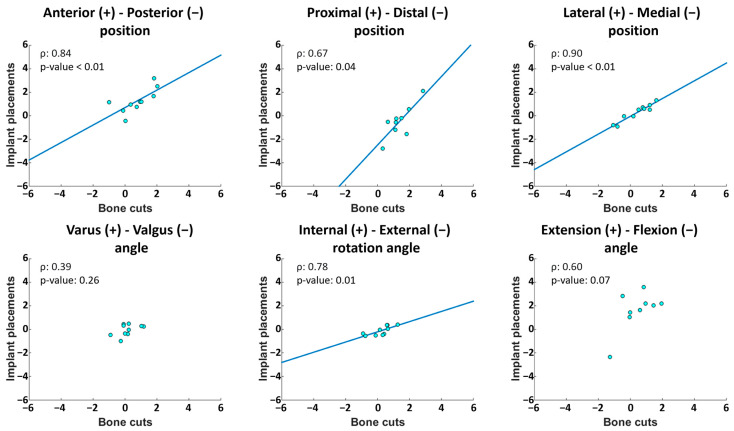
Correlations between femoral bone cut and implant placement errors. When the correlation was statistically significant, the linear regression line was plotted. Data are in mm for positional errors and in degree for angular errors. ρ: Spearman’s rank coefficient of correlation.

**Figure 6:**
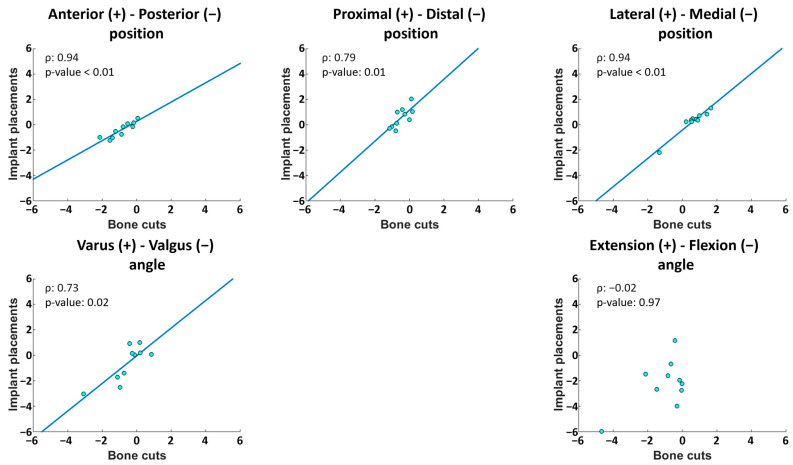
Correlations between tibial bone cut and implant placement errors. When the correlation was statistically significant, the linear regression line was plotted. Data are in mm for positional errors and in degree for angular errors. ρ: Spearman’s rank coefficient of correlation.

Regarding the deviation between bone cuts and implant placements, the RMS values ranged between 0.3–2.0 mm and 0.6–1.9°. Five cases of bias were observed, as illustrated in Figure 4: in the femoral proximal–distal position (*p* = 0.01) and the flexion–extension angle (*p* = 0.04), with implants too distal and extended compared to the cuts (median differences of 1.7 mm and 1.2°, respectively), and in the tibial anterior–posterior (*p* = 0.01), proximal–distal (*p* = 0.01) and medio-lateral (*p* = 0.01) positions with implants too anterior, proximal and medial compared to the cuts (median differences of 0.4 mm, 0.9 mm and 0.3 mm, respectively). A total of 4 outlier deviations were observed out of the 110 individual measurements. Statistically significant correlations between implant placement errors and bone cut errors were observed for four out of the six degrees of freedom in the femur and for four out of the five degrees of freedom in the tibia (ρ ≥ 0.67, *p* < 0.05) (Figure 5 and Figure 6). The statistically non-significant correlations were in the femoral varus–valgus angle (*p* = 0.26), in the femoral flexion–extension angle (*p* = 0.07) and in the tibial flexion–extension angle (*p* = 0.97).

## 4. Discussion

The results showed accurate bone cuts, with RMS errors below 2 mm or 2° and less than 3% outliers (3 out of 110 errors). These results are consistent with a previous study assessing the same system on sawbones [5]. In fact, the median errors differed by less than 0.6 mm or 0.8° between both studies that used distinctive experimental settings, suggesting that the bone cut errors reported in these two studies should indeed correspond to the real accuracy ranges of the system. 

The accuracy of implant placements was in the same range as the bone cut accuracy for nine out of the eleven degrees of freedom (RMS errors under 2 mm or 2°, 2.2% of outliers). These results agreed with a prior study evaluating the same robotic system with patients [6]. In the two other degrees of freedom, femoral and tibial flexion–extension, the RMS errors were 2.9° with 20% outliers. This lower accuracy seemed attributable to the implantation process, since the accuracy of the bone cuts was not markedly lower in these two degrees of freedom than in the others. Interestingly, larger implant placement errors in flexion–extension were not reported in the prior study evaluating the TSOLUTION ONE system [6]. It is therefore possible that the cut–implant deviations vary among implant designs and/or implantation techniques. This possibility is supported by the literature, as other studies evaluating the angular accuracy of TKA performed with image-free navigation systems have also reported larger cut–implant deviations in the sagittal plane [7,8,9]. Further research assessing the error of bone cuts and of implant placements simultaneously, as in the present study, are thus notably encouraged to identify preferable prosthesis designs or implementation techniques. 

A novelty of this study was to characterize the cut–implant deviations both in terms of positions and angles. Deviations between bone cuts and implant placements were generally low, with RMS errors below 2 mm or 2° and no outliers observed in eight of the eleven degrees of freedom. Furthermore, statistically significant correlations were found between bone cut and implant placement errors in seven of these eight degrees of freedom, highlighting the importance of accurate bone cuts for accurate implant placements. Nevertheless, outliers in the cut–implant deviations were observed for the remaining three degrees of freedom, specifically in the tibial flexion–extension angle (10% of outliers), consistent with the literature [7,9], as well as in the femoral proximal–distal position and the flexion–extension angle (20% and 10% of outliers, respectively), for which no data were found in the literature. The limited access to the posterior region of the knee due to the anterior surgical exposure could have contributed to these larger deviations, as the posterior aspect of the implants might have been more difficult to impact. Altogether, these results agree with previous studies which have reported that, despite accurate bone cuts, cement thickness and impaction can cause implant placement errors and induce malalignment [7,19,20]. Therefore, it appears essential to perform the implantation process with caution, particularly regarding the proximal–distal positions and flexion–extension angles. In the future, it might also be possible to reduce the cut–implant deviations by improving implant designs and implantation instrumentations.

Since biases were observed in this study, and it is noteworthy to wonder whether different command of the robotic device could limit them. Although theoretically possible (for example, the femoral bone could be cut more proximally to reduce the proximal–distal implant placement error), a better understanding of the sources of biases would be necessary to ensure that such modifications would actually be beneficial. Indeed, various factors could influence the biases, such as implants, registration points, or calibration of the robotic system, and modifying the command based on one situation could be harmful in other situations. Furthermore, the biases were relatively small and not the sole source of errors. For example, the biases accounted only for a portion of the outlier errors that were observed, up to 3.3 mm and 6.3°. Consequently, further investigations are necessary to understand the sources of both the biases and the outliers. Clarifying this could suggest ways to reduce the bone cut and implant placement errors by acting on the robot or on another aspect of TKA.

Compiling data from two systematic reviews on the accuracy of robotic-assisted systems [21,22] enabled the identification of five cadaveric studies with variables of interest similar to those investigated in the present study [10,11,12,23,24]. Of course, the results in all these studies cannot be rigorously compared, due to variations in experimental aspects (e.g., robotic approach, number and condition of the knees, and assessment methods), and it was not an objective of the present study to compare systems. Nevertheless, it is interesting to put side by side all these results, keeping in mind the variations in experimental aspects. Doing so suggested quite consistent results among the studies, with mean or median errors ranging from 0.0° to 1.3° for femoral and tibial bone cut accuracy (current study: 0.1° to 0.8°) and mean or median errors ranging from 0.1° to 2.0° for femoral and tibial implant placement (current study: 0.1° to 2.1°). Consequently, the current results and those from prior studies, support the potential of robotic-assisted systems for accurate bone cuts and implant placements in TKA.

Several limitations of the present study should be outlined. First, the implants used in this study were nylon, a material that differs structurally from conventional implants, and this could have affected the implantation procedures. However, the use of nylon implants reduced the artefacts in CT scans, enabling more accurate measurement of bone cuts and implant placements. Second, the use of cadaveric specimens led to different bone quality and reduced the mental pressure compared to TKA performed on patients, which could influence the results. Nonetheless, the use of cadaveric samples allowed for the insertion of metallic beads in the bones and the use of nylon implants, which contributed to more accurate assessments. Third, only one type of implant was used. A different prosthesis model could yield different results mainly for implant placement errors and cut–implant deviations. Therefore, further research will be necessary to evaluate the possibility of generalizing the findings to other types of implants. Lastly, the sample size was small, and limited information was available concerning the cadaveric specimens due to ethical and regulatory considerations. A larger population, with an extensive characterization of the specimens, for example, including demographic or structural data, could allow for more specific assessment of the errors and methods, thus highlighting possible areas of improvement. Including a wider panel of surgeons and a control group with conventional surgery could also contribute to a deeper understanding. While these perspectives are attractive, it is noteworthy that the study protocol was adapted to the present objectives and consistent with previous cadaveric studies assessing bone cut and implant placement errors in TKA [10,11,12,23]. 

## 5. Conclusions

This study confirmed the capacity of the TSOLUTION ONE system to achieve accurate bone cuts, both in terms of positions and angles. Moreover, the implant placement accuracy was in the same range as the bone cut accuracy, except in the femoral and tibial flexion–extension angles. This decrease in accuracy could be due to the implantation process, which was the only step occurring between bone cuts and implant placements. Another notable finding of this study concerns the cut–implant deviations, which indicated that accurate bone cuts were necessary for accurate implant placements. But this was not enough; the results also highlighted the importance of the implantation and the possibilities to improve the accuracy of the implant placements by acting on factors such as cement thickness, impaction, or knee exposure. While this study confirmed that robotic-assisted systems can achieve accurate bone cuts and implant placements in TKA, further studies will be required to determine the relationships between these accuracies and the clinical outcomes.

## Data Availability

The data are not publicly available due to regulatory provisions.

## Appendix A

**Table A1:** Positional and angular errors as well as deviations for the femur (*n* = 10 knees).

	Femur					
	Anterior (+)– posterior (−) position	Proximal (+)– distal (−) position	Lateral (+)– medial (−) position	Varus (+)– valgus (−) angle	Internal (+)– external (−) rotation angle	Extension (+)– flexion (−) angle
**Bone cut errors**						
Median {IQR}	0.84 {1.47}	1.16 {0.62} *	0.64 {1.37}	0.08 {0.33}	0.37 {0.66}	0.71 {1.37}
RMS	1.19	1.52	0.96	0.58	0.66	1.55
Percentage of outliers (%)	0	0	0	0	0	10
**Implant placement errors**						
Median {IQR}	1.17 {0.73} *	−0.55 {0.83}	0.50 {0.73}	0.10 {0.70}	−0.20 {0.72}	2.12 {1.21}
RMS	1.60	1.32	0.73	0.46	0.39	2.92
Percentage of outliers (%)	10	0	0	0	0	20
**Cut** **–** **implant deviations**						
Median {IQR}	0.37 {0.53}	−1.70 {0.75} *	−0.18 {0.29}	−0.31 {1.04}	−0.31 {0.57}	1.16 {1.52} *
RMS	0.89	2.02	0.32	0.56	0.55	1.80
Percentage of outliers (%)	0	20	0	0	0	10
ρ (*p*-value)	0.84 (<0.01)	0.67 (0.04)	0.90 (<0.01)	0.39 (0.26)	0.78 (0.01)	0.60 (0.07)

The values in this table indicate where the bone cuts or implant placements were compared to the planning or compared to the bone cuts. For example, a positive anterior–posterior error indicates an implant that was too anterior compared to the planning; similarly a negative varus–valgus deviation indicates an implant that was too valgus compared to the cut. Median, IQR and RMS data are in mm for positional errors and deviations and in degree for angular errors and deviations. IQR: interquartile range; RMS: root-mean-square; ρ: Spearman’s rank coefficient of correlation. *: Statistically significantly different from zero (*p* ≤ 0.05).

**Table A2:** Positional and angular errors as well as deviations for the tibia (*n* = 10 knees).

	Tibia				
	Anterior (+)– posterior (−) position	Proximal (+)– distal (−) position	Lateral (+)– medial (−) position	Valgus (+)– varus (−) angle	Flexion (+)– extension (−) angle
**Bone cut errors**					
Median {IQR}	−0.83 {1.03} *	−0.56 {0.72}	0.69 {0.43}	−0.33 {1.01}	-0.54 {1.14} *
RMS	1.10	0.66	0.99	1.15	1.73
Percentage of outliers (%)	0	0	0	10	10
**Implant placement errors**					
Median {IQR}	−0.33 {0.96}	0.60 {1.12}	0.41 {0.39}	0.07 {1.80}	−2.09 {1.22} *
RMS	0.69	0.94	0.93	1.49	2.86
Percentage of outliers (%)	0	0	0	10	20
**Cut–implant deviations**					
Median {IQR}	0.43 {0.30} *	0.87 {0.64} *	−0.31 {0.28} *	0.00 {0.98}	−1.24 {1.91}
RMS	0.56	1.16	0.42	0.80	1.89
Percentage of outliers (%)	0	0	0	0	10
ρ (*p*-value)	0.94 (<0.01)	0.79 (0.01)	0.94 (<0.01)	0.73 (0.02)	−0.02 (0.97)

The values in this table indicate where the bone cuts or implant placements were compared to the planning or the bone cuts. For example, a negative anterior–posterior error indicates an implant that was too posterior compared to the planning, similarly a positive proximal–distal deviation indicates an implant that was too proximal compared to the cut. Median, IQR and RMS data are in mm for positional errors and deviations and in degree for angular errors and deviations. IQR: interquartile range; RMS: root-mean-square; ρ: Spearman’s rank coefficient of correlation. *: Statistically significantly different from zero (*p* ≤ 0.05).
